# Impaired Oligodendrocyte Development Following Preterm Birth: Promoting GABAergic Action to Improve Outcomes

**DOI:** 10.3389/fped.2021.618052

**Published:** 2021-02-04

**Authors:** Julia C. Shaw, Gabrielle K. Crombie, Hannah K. Palliser, Jonathan J. Hirst

**Affiliations:** ^1^School of Biomedical Sciences and Pharmacy, University of Newcastle, Newcastle, NSW, Australia; ^2^Mothers and Babies Research Centre, Hunter Medical Research Institute, New Lambton Heights, NSW, Australia

**Keywords:** preterm (birth), oligodendrocyte, GABA, glutamate, neurosteroids

## Abstract

Preterm birth is associated with poor long-term neurodevelopmental and behavioral outcomes, even in the absence of obvious brain injury at the time of birth. In particular, behavioral disorders characterized by inattention, social difficulties and anxiety are common among children and adolescents who were born moderately to late preterm (32–37 weeks' gestation). Diffuse deficits in white matter microstructure are thought to play a role in these poor outcomes with evidence suggesting that a failure of oligodendrocytes to mature and myelinate axons is responsible. However, there remains a major knowledge gap over the mechanisms by which preterm birth interrupts normal oligodendrocyte development. *In utero* neurodevelopment occurs in an inhibitory-dominant environment due to the action of placentally derived neurosteroids on the GABA_A_ receptor, thus promoting GABAergic inhibitory activity and maintaining the fetal behavioral state. Following preterm birth, and the subsequent premature exposure to the *ex utero* environment, this action of neurosteroids on GABA_A_ receptors is greatly reduced. Coinciding with a reduction in GABAergic inhibition, the preterm neonatal brain is also exposed to *ex utero* environmental insults such as periods of hypoxia and excessive glucocorticoid concentrations. Together, these insults may increase levels of the excitatory neurotransmitter glutamate in the developing brain and result in a shift in the balance of inhibitory: excitatory activity toward excitatory. This review will outline the normal development of oligodendrocytes, how it is disrupted under excitation-dominated conditions and highlight how shifting the balance back toward an inhibitory-dominated environment may improve outcomes.

## Introduction

The incidence of preterm birth has stubbornly remained at ~8%, with the majority (~74%) of these deliveries falling into the moderate to late preterm range (32–36 weeks of gestation) (1). Although short-term outcomes are good, these neonates have markedly disrupted brain development that persists into later life (2). In addition to major preterm birth related disorders, such as cerebral palsy and bronchopulmonary dysplasia, there is a well-established body of evidence supporting the notion that preterm infants born in the moderate to late range are also much more likely to develop neurodevelopmental morbidities and learning disorders that become apparent at around school age (3–9). These disorders include internalizing disorders (such as anxiety and depression), inattentive attention deficit hyperactivity (ADHD) disorder and poor social skills (10). These disorders can be observed from pre-school age through to adulthood (10), but importantly, often occur in the absence of overt brain injury at the time of birth. Development of these disorders leads to significant socioeconomic burden for these individuals as well as for their families and the healthcare system (11). Thus, there is an urgent need to develop therapeutic strategies to reduce these negative effects of moderate to late preterm birth and we propose that this needs to be done in the early neonatal period.

Moderately to late preterm born neonates frequently already have, or will develop, subtle deficits in white matter tracts not visible on routine MRI (1, 12, 13) which persist beyond the time of full term, and despite further post-term development of myelination, behavioral disorders emerge in later life (1, 2, 7, 13). This review will examine the processes surrounding oligodendrocyte development, specifically in the late gestation fetus, and how premature exposure to the *ex utero* environment disrupts this process. We will also cover studies showing moderate to late gestation is characterized by an inhibitory tone in the developing brain that is subsequently lost following preterm birth. This inhibitory tone is maintained by the placentally derived neurosteroid, allopregnanolone (5α-pregnane-3α-ol-20-one) and its actions on the γ-aminobutyric acid a (GABA_A_) receptor, which plays a key role in oligodendrocyte development *in utero* (14). Finally, we will also discuss some approaches that promote maturation of the oligodendrocyte lineage and myelination in the newborn brain resulting in improved neurodevelopmental outcomes.

## Oligodendrocyte Development

**Figure 1:**
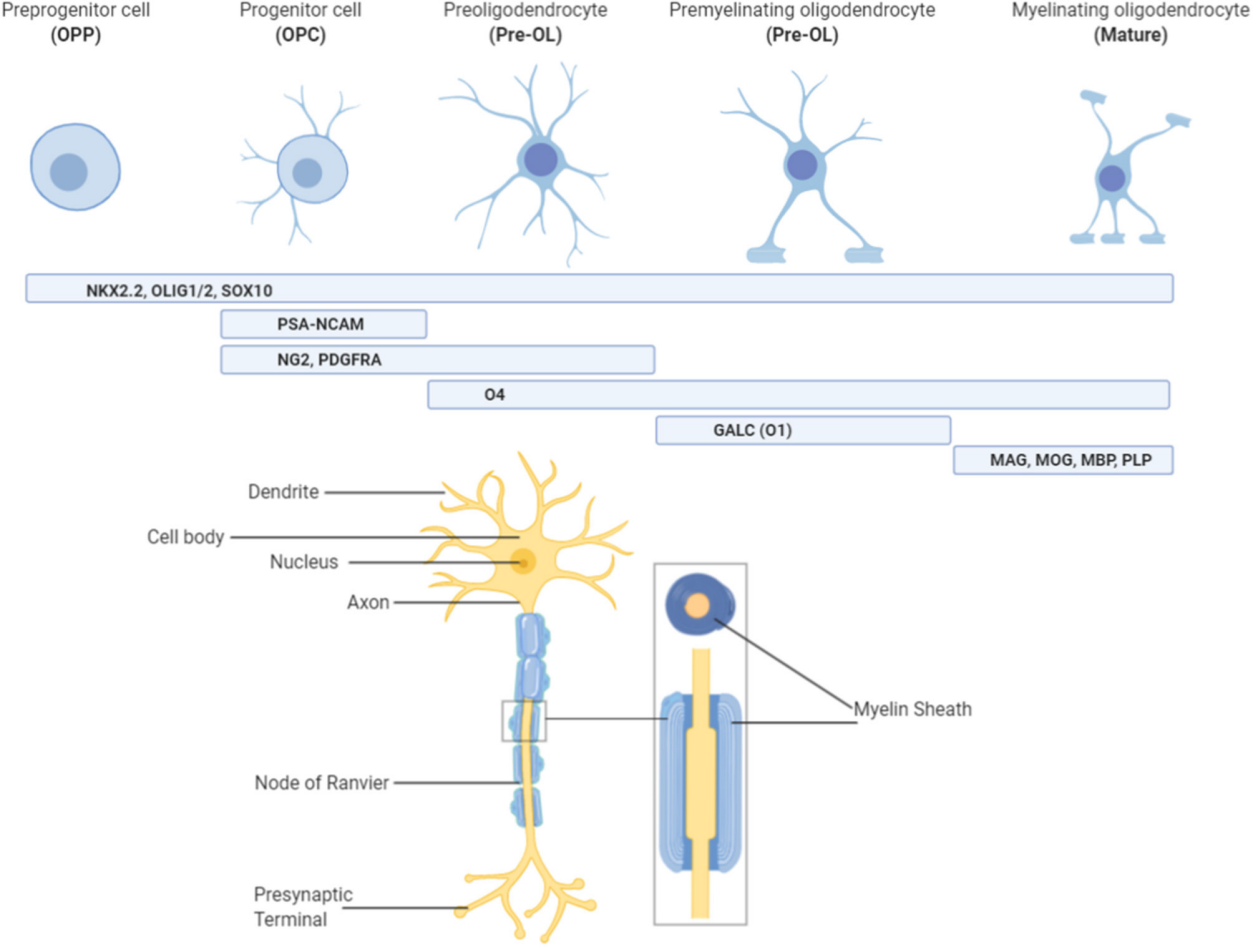
Characterizing oligodendrocytes throughout the lineage. Oligodendrocytes originate from pre-progenitor (OPP) neural cells and are committed to the oligodendrocyte pathway under the influence of NKx2.2, Olig1/2, and Sox10. Once committed to the pathway, stage specific markers of oligodendrocytes allow for characterization of the lineage. Progenitor (OPC) and pre-oligodendrocytes (Pre-OL) feature stage-specific growth factor receptors (platelet derived growth factor receptor alpha; PDGFRα), surface antigens (neural/glial antigen 2; NG2) and cell adhesion molecules (polysialylated-neural cell adhesion molecule; PSA-NCAM), whilst premyelinating oligodendrocytes possess enzymes for lipid synthesis (galactocerebrosidase; GalC/O1). Finally, myelinating oligodendrocytes are characterized by the presence of myelin proteins such as myelin-associated glycoprotein (MAG), myelin basic protein (MBP), myelin oligodendrocyte glycoprotein (MOG), and proteolipid protein (PLP). Oligodendrocytes at this final stage of the lineage are the only ones capable of producing myelin and must also have contact with neuronal axons to perform this role. Figure created with BioRender.com.

Oligodendrocytes progress through a number of morphological and functional changes, from their origins as neural stem cells, to mature oligodendrocytes capable of myelin production (Figure 1). This is a highly regulated process that has already been described in detail elsewhere (15, 16). Briefly, neural stem cells commit to the oligodendrocyte lineage and become oligodendrocyte precursor cells (OPCs) under the influence of transcription factors including *Olig1/2, NKX2.2*, and *Sox10* (16, 17). From here, OPC expansion is heavily influenced by growth factors such as platelet derived growth factor (*PDGF*), which promote proliferation but inhibit differentiation (16, 18, 19). This ensures that a large pool of OPCs are created before they are committed to differentiation, which is an irreversible process. Thus, oligodendrocyte differentiation is driven by a loss of this “inhibition to differentiate” environment, likely by promoting expression of microRNAs that prevent transcription of differentiation inhibitors (16, 20, 21). Once OPCs have matured into pre-oligodendrocytes (pre-OLs) they differentiate under the control of a number of transcription factors, but one of the most crucial is known as myelin regulatory factor (*Myrf*) (16, 22). Expression of *Myrf*, and its interaction with *Sox10*, in differentiating oligodendrocytes induces the activation of genes encoding lipid structural proteins such as phosphodiesterase Enpp6, and thus enables the production of myelin (23). Deletion or inactivation of the *Myrf* gene prevents the generation of differentiated oligodendrocytes (23), without affecting pre-existing oligodendrocytes or myelin, and is associated with a subsequent impairment of learning ability, highlighting the integral role that this transcription factor, and oligodendrocyte development in general, plays in normal neurodevelopment.

Oligodendrocyte development is also driven by extracellular signals (16), such as endogenous glucocorticoids and neurotransmitters. A study in adult mice found that oligodendrocyte progenitors and mature oligodendrocytes express glucocorticoid receptors, leading the authors to suggest that glucocorticoids are involved in the differentiation processes of oligodendrocytes (24). Furthermore, the presence of steroid hormone cofactors that increase the transcriptional activity of glucocorticoid receptors is expressed in oligodendrocyte progenitor cells but not mature oligodendrocytes, thus strengthening the notion that glucocorticoids play a role in driving differentiation (24). It is important to note that whilst normal physiological levels of glucocorticoids, such as cortisol, are required for oligodendrocyte development, levels above normal may be detrimental due to the high expression of these receptors. Meanwhile, the neurotransmitter GABA is also involved in oligodendrocyte differentiation and myelination through activation of GABA_A_ and GABA_B_ receptors (25). In a hypoxic mouse study, addition of GABAergic drugs tiagabine and vigabatrin increased the number of mature oligodendrocytes, whilst addition of a GABA_A_ receptor antagonist prevented this (26). Additionally, it was shown in a purified rat oligodendrocyte progenitor culture that addition of GABA accelerates oligodendrocyte differentiation by promoting branching and myelin protein expression (27). Importantly, these effects can be blocked by a GABA_B_ receptor-specific antagonist (27). Furthermore, GABAergic neurons establish synaptic connections with oligodendrocytes to control differentiation and migration and ultimately induce the wrapping of axons (25).

The translation of myelin proteins such as myelin–associated glycoprotein (MAG), myelin oligodendrocyte glycoprotein (MOG), myelin basic protein (MBP), and myelin proteolipid protein (PLP) is reliant on contact with axons for the wrapping of myelin to occur (16, 28). Despite OPCs appearing in the fetal brain at ~10 weeks of gestation, it is not until ~30 weeks of gestation when the myelination of axons begins (16). Therefore, oligodendrocyte development may be markedly impacted by preterm birth during these final stages of maturation and myelination. Importantly, preclinical studies show that myelin proteins, such as MBP and PLP, are reduced in animals exposed to moderate to late gestation perinatal insults such as growth restriction and preterm delivery, whilst those expressed at earlier stages of the lineage are unaffected (29–34). This is to be expected given the developmental timeline, but critically, these relative reductions persist throughout life. The key question then becomes, how does preterm birth prevent this expansive pool of OPCs and pre-OLs from maturing and producing myelin? We propose the *ex utero* environment plays a crucial role in ongoing deficits as the biological immaturity of the brain at the time of birth predisposes preterm neonates to respond poorly to *ex utero* insults such as increased cortisol, and periods of hypoxia, all in the absence of protective placental neurosteroid support. Furthermore, while oligodendrocyte development is heavily influenced by transcription factors and growth factors (16), it is also regulated by extracellular signals (16), hence a perturbation in these signals may affect the development of the lineage.

### Perturbations to Oligodendrocyte Development After Preterm Birth

Following preterm birth, the newborn is exposed to the *ex utero* environment earlier than if it had remained *in utero* until term. Oligodendrocytes are highly sensitive to adverse conditions and are frequently injured by chemical and mechanical damage, which can occur following early delivery and the consequent premature exposure to the *ex utero* environment (35, 36). Substantive evidence indicates that ex-premature children experience impaired learning, and a loss of myelination is evident suggesting a causal pathway (13, 37–41). In infants born <27 weeks, diffuse microstructure alterations are observed on fractional anisotropy at term equivalence age in regions such as the corpus callosum and frontal cortex white matter when compared to term controls (38). Similarly, myelination deficits can be observed at term equivalence age in infants born <30 weeks and diagnosed with periventricular leukomalacia (PVL) (42). As the gestational age at the time of birth increases, these white matter alterations become much harder to detect using routine imaging techniques whilst poor learning and behavioral outcomes remain evident (13, 43).

Post-mortem human studies have identified the relatively subtle effect of moderate to late preterm birth on the oligodendrocyte population, with the expression of Olig2-labeled cells significantly increased in areas with increased astrocytes, indicating injury (44). Additionally, myelination is sparse in these brains (44). Double-labeling of the Olig2 cells with stage-specific markers of the lineage revealed that the pre-oligodendrocyte population was increased in areas of injury, whilst the immature oligodendrocyte population was unaffected (44). This distinctive feature of moderate to late preterm birth-related white matter injury highlights that there is an imbalance in the oligodendrocyte lineage following preterm birth, with an increased percentage of pre-oligodendrocytes and a lower percentage of immature oligodendrocytes (44). In addition, whilst total neuron number is unaffected in areas indicative of white matter injury, the number of GABAergic neuronal cells is significantly decreased (45). It is possible that this loss of GABAergic neurons may contribute to the “arrest” in oligodendrocyte maturation due to a lack of the synaptic coupling with oligodendrocytes that induces myelin production and wrapping of axons, or due to the loss of GABA production which may lead to a shift away from an inhibitory dominant environment, and a shift toward an excitatory (glutamate) dominant environment. Thus, the primary characteristic of oligodendrocyte failure in the preterm brain is suggested to be an expansion of the pre-oligodendrocyte population, which is then unable to develop further, resulting in a net loss of ongoing myelination (39, 46, 47). There remains a major knowledge gap over the mechanisms that lead to this failure and how long it may persist for, and thus there is a current lack of effective therapies to combat preterm-birth related deficits in myelination. Below we present evidence to suggest that *ex utero* factors in the immediate postnatal period such as increased cortisol, periods of hypoxia, increases in excitability and loss of nurturing neurosteroids impact upon the overall biological immaturity of the preterm brain to result in a failure of oligodendrocytes to mature.

## Importance of *in utero* Neurosteroid Concentrations

Neurosteroids are steroid hormones that not only protect the fetal brain but also form a key neuromodulatory system that regulates excitability and development during at least the second half of gestation in long gestation species including human, sheep and guinea pigs (14, 33, 48–50). In these species, progesterone production by the placenta provides precursors for the production of these neuroactive metabolites that influence the fetal brain (14, 50, 51). This placenta-brain interaction is critical in maintaining fetal brain excitability and development at least in late gestation. Allopregnanolone is the key neurosteroid during gestation with levels supported by the high level of placental progesterone production and metabolism (52, 53). This leads to allopregnanolone levels in the fetal circulation and brain that are markedly higher than in the neonate after birth and in the adult brain (33, 54). There is a marked decline in allopregnanolone levels following the fetal to neonatal transition in both preterm and term neonates (33). Therefore, preterm birth leads to a premature decline in allopregnanolone levels with animal studies showing this contributes to the reduced myelination that is associated with adverse patterns of behavior in the offspring (31, 33, 34). Importantly, although replacement with allopregnanolone analogs improves outcomes (55), progesterone treatment of neonates while raising allopregnanolone levels in the fetal circulation, does not appear to fully reverse the adverse effects of preterm birth on brain development (31). This may be because local levels in the brain are not adequately elevated or that progesterone may be metabolized to other steroids, in this instance cortisol (31), that are not effective in improving outcomes. These observations suggest that both progesterone production by the placenta and its metabolism to allopregnanolone is required to produce the nurturing steroid environment of the fetal brain.

### Allopregnanolone Promotes Oligodendrocyte Development

Allopregnanolone is one of the major agonists of the GABA_A_ receptor and elevated gestational levels in the fetal brain markedly increase the activity of inhibitory GABAergic pathways. Stimulation of the GABA_A_ receptor in early gestation invokes an excitatory action, however this undergoes a switch to inhibitory (56). In species such as the guinea pig, non-human primate, and human this switch occurs at around 0.6 of gestation (57, 58), however in other rodent species, such as the rat and mouse, the switch occurs postnatally (59, 60). The switch is controlled by the developmentally regulated change in the expression of two co-transporters, the potassium chloride co-transporter (KCC2) and the sodium potassium chloride co-transporter (NKCC1), which control the influx and efflux of chloride (61, 62). In guinea pigs, non-human primates, and humans the marked decline in allopregnanolone after birth results in a consequent fall in GABAergic inhibition (33, 48). Therefore, it can be hypothesized that preterm birth leads to reduced GABA-mediated inhibitory tone, which may lead to reduced trophic drive for ongoing myelination. The sensitivity of GABA_A_ receptors to allopregnanolone and other agonists is determined by the subunit composition of the receptors (63). The presence of α4–6 and δ-subunits in the GABA_A_ receptor complex increases sensitivity to neurosteroid binding (50) and therefore receptors containing these subunits are important in driving trophic processes. Glial cells, and importantly oligodendrocytes, express GABA_A_ receptors that are stimulated by extrasynaptic GABA released from nearby presynaptic terminals, with this stimulation strengthened by the co-binding of allopregnanolone (64). The GABA_A_ receptor subtypes expressed in oligodendrocytes remains unclear and requires investigation, however allopregnanolone has been shown to stimulate both oligodendrocyte precursor and mature cells (65), supporting a gliotrophic interaction with GABA_A_ receptors on these cells. Together these findings suggest that the supportive effects attributed to GABAergic pathways in the fetus are mediated by extrasynaptic allopregnanolone-sensitive receptors (65–67), supporting the contention that GABAergic pathways have a major role in oligodendrocyte development that is prematurely lost following preterm birth. Thus, we suggest that GABA_A_ receptor action has at least two key stimulatory roles in late gestation, (i) increasing maturation of oligodendrocytes and (ii) enhancing myelin production by mature cells.

## Role of Environmental Insults in Failure of Oligodendrocytes to Mature

### Increased Cortisol

Neonatal intensive and special care units are inherently, but unavoidably, stressful for the preterm neonate. Repeated painful but necessary medical procedures, such as drug administration, heel prick blood collection, and respiratory management, as well as over-stimulation due to noise and light, have the potential to increase circulating cortisol concentrations in the preterm neonate (10, 68, 69). This increased cortisol in the neonatal period may be a key contributor to ongoing deficits in oligodendrocyte development, with evidence pointing to excess glucocorticoid-induced damage to oligodendrocytes that impedes their myelinating capability (70). Recently published embryonic rat spinal cord and cerebral cortex *in vitro* studies utilizing corticosterone, dexamethasone, and the glucocorticoid-receptor antagonist Mifepristone shows that prolonged exposure to glucocorticoids induces a dose-dependent reduction in myelination, which is prevented by Mifepristone (70). Interestingly, infection and chorioamnionitis associated utero-placental inflammation, a major cause of preterm birth, may also increase cortisol exposure. Placental cell culture studies have shown that infection-induced cytokines suppress placental 11β-hydroxysteroid dehydrogenase type 2 (HSD2) expression which would increase the passage of cortisol to the fetus (71). This increase in cortisol exposure may suppress allopregnanolone synthesis and potentiate the effects of inflammation on oligodendrocytes by lessening allopregnanolone-induced protection. Alternatively, some earlier studies have shown that neonatal treatment with lipopolysaccharide induced inflammation and raised allopregnanolone concentrations in the brain (72). The mechanism involved in this response is unclear and further studies of the potentially protective neurosteroid responses to infection are warranted.

**Figure 2:**
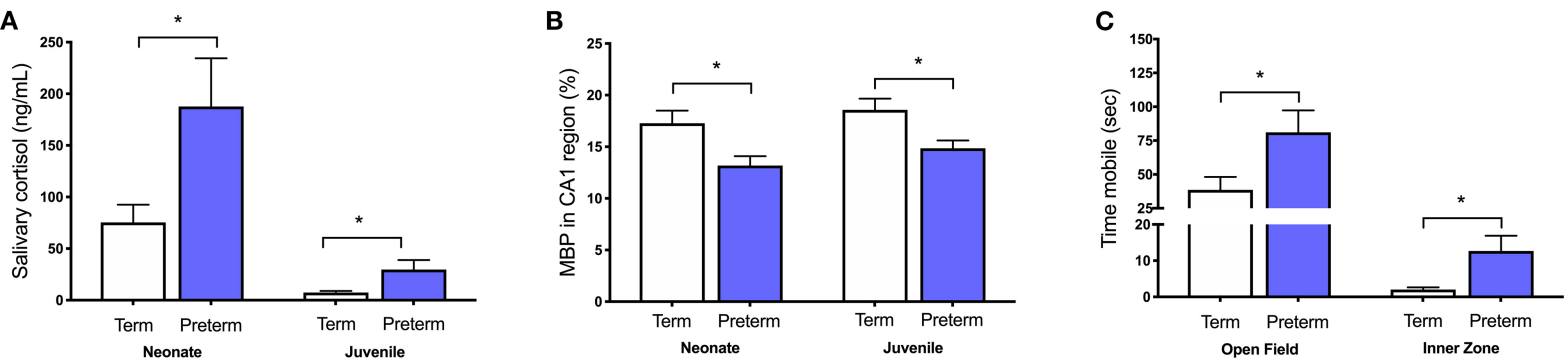
Increased cortisol in the preterm neonate is associated with poor outcomes. Male guinea pigs born preterm (GA62; blue bars) have **(A)** higher salivary cortisol concentrations as a neonate (24 h old) and as a juvenile (corrected postnatal day 25), **(B)** decreased area coverage of myelin basic protein (MBP) in the CA1 region of the hippocampus, and **(C)** exhibit hyperactive behavior by spending more time mobile in the open field and inner zone when compared to term born (GA69; white bars) age-matched controls. Adapted from (34, 77). *p < 0.05, *n =* 4–10.

We have also shown *in vivo* that increases in cortisol are associated with numerous other perinatal compromises, including intrauterine growth restriction, maternal stress exposure, pharmacological inhibition of allopregnanolone synthesis, and preterm birth (31, 32, 34, 73–79). In each of these cases there is a clear relationship between increased cortisol, decreased allopregnanolone, and mature myelin loss in vulnerable regions such as the hippocampus and cerebellum that are developing rapidly during the period of exposure. Furthermore, these stressful perinatal environments have long-lasting effects as we have also demonstrated that childhood-equivalent age behavior is affected by these *in utero* and immediate postnatal period exposures to abnormally high cortisol. Specifically, moderate to late gestation maternal stress exposure in guinea pigs increases maternal cortisol concentrations and reduces myelination in the fetal hippocampus (73). This deficit in myelination, as evidenced by reduced MBP immunostaining, persists until at least childhood-equivalent age, suggesting an ongoing loss of oligodendrocytes or a maturational arrest in their development (75, 76). Additionally, guinea pigs that are born preterm have increased salivary cortisol concentrations in the first 24 h of life, which remains elevated for males until at least childhood-equivalent age. This is associated with hyperactive behavior and deficits in hippocampal myelination (Figure 2) (34, 77).

Low neurosteroid environments, such as following preterm birth, and increased glucocorticoid action appear to have complex interactions. For example, in human hepatocyte cultures treatment with finasteride increased the action of cortisol on the glucocorticoid receptor, whilst overexpressing the enzyme responsible for neurosteroid synthesis (5α-Reductase type 1) dampened the effect of cortisol in these cultured cells (80). *In vivo* we have shown that pharmacologically increased plasma cortisol in preterm delivered male guinea pig neonates at term equivalence age is associated with an exacerbated reduction at the early and late stages of the oligodendrocyte lineage in the cerebellum. This was demonstrated by decreased Olig2, PDGFRα, and PLP protein expression (31). Furthermore, in prenatally stressed rats, exaggerated corticosterone responses to immune challenge with IL-1β were prevented with allopregnanolone pre-treatment, suggesting an attenuation of the stress response by neurosteroids (81). Repeated maternal betamethasone administration in late gestation guinea pigs also negates the protective effect of neurosteroids by reducing their synthesis in the placenta (82). A single course of betamethasone is standard clinical practice when there is a risk of preterm birth, as the glucocorticoid exposure is required to accelerate fetal lung development. Despite this finding in the guinea pig placenta, in humans exposure to a single course of betamethasone is not associated with an alteration in the neurosteroid synthesis enzymes 5α-reductase type 1 and 2 in the placenta (83), presumably due to the repeated vs. single course administration. Additionally, a study in rabbits using a single course of betamethasone based on the recommended human dose revealed no adverse effects of betamethasone on GABAergic and glutamatergic neurogenesis (84). However, a recent population-based retrospective cohort study in Finland has identified a significant increase in behavioral disorders in children that were exposed to a single course of betamethasone *in utero* (85). The significant increase was evident in both the preterm and term populations that were exposed to betamethasone, compared to age-equivalent children that were not exposed. This disconcerting finding highlights the detrimental effect that inappropriate glucocorticoid exposure in the perinatal period may have on later neurodevelopment.

These long-lasting impacts of glucocorticoids may be mediated by epigenetic changes in the immature preterm brain. Stress in pregnancy for example has been shown to increase methylation of the *GAD1* gene, responsible for the conversion of glutamate to GABA, in hippocampal GABAergic neurons (86). A hyperactive phenotype was observed in these same mice at juvenile age but was prevented by Clozapine, an anti-psychotic but also a DNA-demethylation drug, supporting the link between stress-induced methylation changes in the brain and long-term behavior (87). Glucocorticoids have also been shown to affect histone acetylation, with synthetic glucocorticoid exposure in male guinea pig fetuses increasing acetylation in the hippocampus, suggesting that transcriptionally silenced genes are possibly becoming activated following inappropriate glucocorticoid exposure (88, 89). Furthermore, ill-timed glucocorticoid exposure in the fetal guinea pig induces permanent changes in functioning of the hypothalamic-pituitary-adrenal (HPA) axis and behavior that are passed down to the F1 offspring, again suggesting a role of epigenetic modifications (90, 91). However, whether these effects specifically target the transcription of regulators of the oligodendrocyte lineage or play a role in their failure to produce myelin following preterm birth is not clear and warrants investigation.

### Periods of Hypoxia

Perinatal hypoxia, due to adverse events during labor or as a result of inadequate lung maturation for example, is relatively common in the preterm delivered population compared to term-born neonates (92). There is no doubt that hypoxia contributes to white matter injury in the preterm neonate (92). In particular, there is an extensive number of studies, in both human and animal models, supporting the notion that pre-OLs in particular are highly susceptible to hypoxic-related cell death following activation of caspase-3 (26, 47, 93, 94). Conversely, OPCs exhibit a robust response to hypoxia whereby the OPC pool is expanded as compared to the depletion of pre-OL cells (26, 39). This response means that a replenished population of pre-OL cells is created, but these new pre-OLs then fail to mature further, ultimately resulting in a net loss of myelin (39). Double labeling NG2 positive oligodendrocytes with the proliferation marker Ki67 in the neonatal rat cerebellum identifies the OPC pool of oligodendrocytes as those expanding in response to hypoxia, whilst the expression of mature oligodendrocyte markers was reduced for at least 20 days (26). Another neonatal rat model of hypoxia showed similar findings, with loss of the pre-OL pool by caspase-3 mediated cell death in the acute period, followed by a robust regeneration but a subsequent failure of this new cell population to mature (47). This type of oligodendrocyte injury is seen clinically in white matter lesions of deceased preterm neonates, where there is a lower percentage of immature oligodendrocytes compared to controls (94), and MRI assessment of myelination in children and adolescents born preterm. These studies have identified diffuse white matter microstructure deficits linking this acute loss of preOLs with a long-term reduction in myelin (41).

Damage due to hypoxic ischemic events is strongly linked with an increase in glutamate receptor activation, and a subsequent flow of excess calcium ions into the cell, leading to cell death *in vitro* (95–99). Decreased ATP during hypoxia leads to a reversal of glutamate transporters (100), with the result being an increase in extracellular glutamate release, primarily from astrocytes (99). Thus, there is increased glutamate which can then readily activate a-amino-3-hydroxy-5-methyl-isoxazolepropionic acid (AMPA) and kainate receptors located on oligodendrocytes (98, 101, 102), and N-methyl-D-aspartate (NMDA) receptors on myelin sheaths (102–104). In a sheep model of hypoxia, repeated umbilical cord occlusion resulted in marked glutamate efflux in the cerebral white matter where subjects with the greatest increases in extracellular glutamate following occlusion also had brain injury representative of PVL, suggesting a key role of glutamate (105). *In utero*, the fetus is protected from hypoxic periods by placentally-derived GABAergic agonist neurosteroids, such as allopregnanolone, which are much lower in the *ex utero* environment (106–108). The protective action of allopregnanolone has been demonstrated in late gestation fetal sheep following umbilical cord occlusion where allopregnanolone was increased in the fetal brain in response to asphyxia and, importantly, was shown to play a key role in protecting the fetal brain from asphyxia-induced cell death in vulnerable regions including the hippocampus and cerebellum (108). Infusion of finasteride (an inhibitor of allopregnanolone synthesis), in addition to umbilical cord occlusion, significantly increased the amount of caspase-3 mediated cell death in neurons and astrocytes throughout the fetal brain, an effect that was lessened in the presence of normal gestational allopregnanolone concentrations (107). Interestingly, when a double infusion of finasteride and alfaxalone (an allopregnanolone analog) was performed, the effects of finasteride on behavioral activity and cell death were not seen, again highlighting the protective role of neurosteroids (109). Whilst these studies did not investigate the specific effect on oligodendrocytes, subsequent studies performed in the guinea pig show that inhibition of neurosteroid synthesis significantly decreases myelination in the subcortical white matter and experimentally induced growth restriction reduces myelination in the hippocampus (32). Altogether, these late gestation preclinical studies support the protective nature of allopregnanolone and therefore has implications for the preterm neonate where exposure to hypoxic periods often occurs in the relative absence of allopregnanolone.

### Disruption to Sleep-Wake-Like Cycling

As oligodendrocytes produce myelin, their cell membrane expands to eventually support a membrane of 100 times their original size (15, 110). Understandably, this is a very high-energy demanding process, which under normal circumstances would take place whilst the fetus is *in utero* (1, 111, 112). In the case of preterm birth, this process takes place in the stimulating *ex utero* environment. Interestingly, oligodendrocytes of fetal origin are highly sensitive to glucose deprivation, exhibiting a failure to differentiate under these conditions, whilst oligodendrocytes of adult origin are relatively unaffected (113). Marked effects of glucose depletion are observed across the lineage *in vitro*, with low glucose concentrations inhibiting OPC differentiation and migration, reducing cell numbers across the lineage, and preventing myelination (114, 115). Glucose deprivation not only affects development and survival of oligodendrocytes, but also the morphology of surviving cells with reductions in branching and thinning of processes (116). Glucose levels are higher during sleep than wake (117) supporting the importance of the sleep-wake cycle during this period, and the contribution of fetal sleep-like states to promoting myelination.

Microarray results have identified subsets of genes that are differentially expressed in oligodendrocytes depending on sleep or wake state (118–120). During sleep, immature oligodendrocytes have a higher expression of genes associated with cell proliferation, including *Nrg2* (Neuregulin 2) which is essential for OPC proliferation through its activation of *ErbB* (Epidermal growth factor) family receptors (121, 122). Conversely, mature oligodendrocytes have a higher expression of genes related to phospholipid synthesis and myelination, such as *Pllp* (plasma membrane proteolipid) and *Opalin* (Oligodendrocytic myelin paranodal and inner loop protein) during sleep (121). Thus, the fetal sleep-like state, at least partially regulated by high levels of neurosteroids and GABAergic inhibitory activity (14), may be important for proliferation of OPCs and production of myelin by mature oligodendrocytes. Conversely, genes increased during periods of wakeful-like activity have roles in cell differentiation (121), which is consistent with the increase in glutamate during this state (123). Glutamate signaling through AMPA receptors promotes differentiation of oligodendrocytes whilst inhibiting OPC proliferation. This therefore has implications for the preterm neonate, where a loss of neurosteroid-GABA activity and exposure to the *ex utero* conditions interrupts the normal sleep-wake cycle, potentially affecting the expression of “sleep” genes, and contributing to the failure of mature oligodendrocytes to myelinate.

We have demonstrated that the neurosteroid-GABA_A_ interaction regulates excitability in fetal life and has a major role in maintaining the fetal “sleep-like” states. In sheep, treatment with finasteride, an inhibitor of neurosteroid synthesis (5a-reductase type 1 and 2) at ~0.88 of gestation (130 days out of a 147 day pregnancy) triggers an arousal-like and hyperactive state in fetal behavioral patterns (14, 49, 55). Similarly, fetal arousal behavior is also increased following treatment with trilostane, an inhibitor of progesterone synthesis (3β-hydroxysteroid dehydrogenase) and thus subsequent allopregnanolone synthesis (124, 125). Importantly, fetal behavior returns to normal following a subsequent infusion with progesterone (125). Therefore, when a neonate is born premature it is separated from this neurosteroid-rich environment, and the supportive fetal inhibition dominated “sleep-like” state is lost.

## Environmental Insults Lead to Glutamate Excitotoxicity

Glutamate, the main excitatory neurotransmitter of the CNS, is also involved in oligodendrocyte development, but this is limited to the proliferative stage of OPCs where glutamate guides migration through activation of AMPA receptors (65, 126). Conversely, increased glutamate present at the later stages of oligodendrocyte development promotes a rather hostile response. The expression of NMDA, AMPA and kainate receptors on oligodendrocytes makes them especially sensitive to increased extracellular glutamate and subsequent excitotoxic damage following excessive activation of the receptors *in vitro* (96–98, 103). In cultured oligodendrocytes, overactivation of ionotropic glutamate receptors results in an influx of calcium ions into the cell, the generation of reactive oxygen species (ROS) and activation of cell death pathways (95, 127). Interestingly, accumulation of ROS in oligodendrocytes following AMPA receptor overactivation is higher than in neurons (95), highlighting the increased susceptibility of oligodendrocytes to fluctuations in extracellular glutamate which is then further compounded by their inability to resist oxidative stress. Of the three receptor families, AMPA receptors (particularly those lacking the GluR2 subunit) may pose the greatest danger as activation of this receptor family results in the highest influx of calcium ions (95, 96). AMPA receptors undergo a developmental switch from highly calcium permeable GluR2-lacking receptors in early development, to GluR2-containing calcium impermeable receptors postnatally (128). However, it has been suggested that neurological insults can decrease the expression of the GluR2-subunit (129), for example global ischemia increases the expression of genes that suppress GluR2 gene expression, thus increasing the permeability of AMPA receptors to calcium and the risk of excitotoxic cell death (130). Relative protein levels of the AMPA receptor subunits GluR1-GluR4 are differentially developmentally regulated in the human brain from mid gestation through to early childhood (131). Of note, the period where preterm birth may occur (between 25 and 37 weeks) shows low expression of the GluR2 subunit and thus highlights a vulnerable window for excitotoxic damage due to the increased potential for calcium influx (131). Furthermore, it has also been confirmed that AMPA receptors are expressed on developing human oligodendrocytes that populate fetal white matter within this preterm period of 23–32 weeks (132). These authors further demonstrated that addition of an AMPA-kainate receptor antagonist prevents calcium influx and glutamate excitotoxic cell death (132). Thus, these studies suggest that preOLs in particular are very susceptible to glutamate-induced oxidative stress due to the presence of GluR2-lacking AMPA receptors, which is exacerbated by their low expression of antioxidant enzymes and reduced capability to scavenge free radicals (133, 134). These observations are especially noteworthy given that human and animal studies suggest this stage of the lineage is the most adversely affected by preterm birth. Conversely, there is data to suggest mature oligodendrocytes are sensitive to glutamate excitotoxicity. Administration of glutamate in a rat model of spinal cord injury activated cell death pathways in mature oligodendrocytes, as evidenced by increased co-localized labeling of the mature oligodendrocyte cell marker CC1 [also known as adenomatous polyposis coli (APC)] with caspase-3 (135). Damage was most evident 6 h following administration but persisted for at least 1 week after the glutamate exposure.

**Figure 3:**
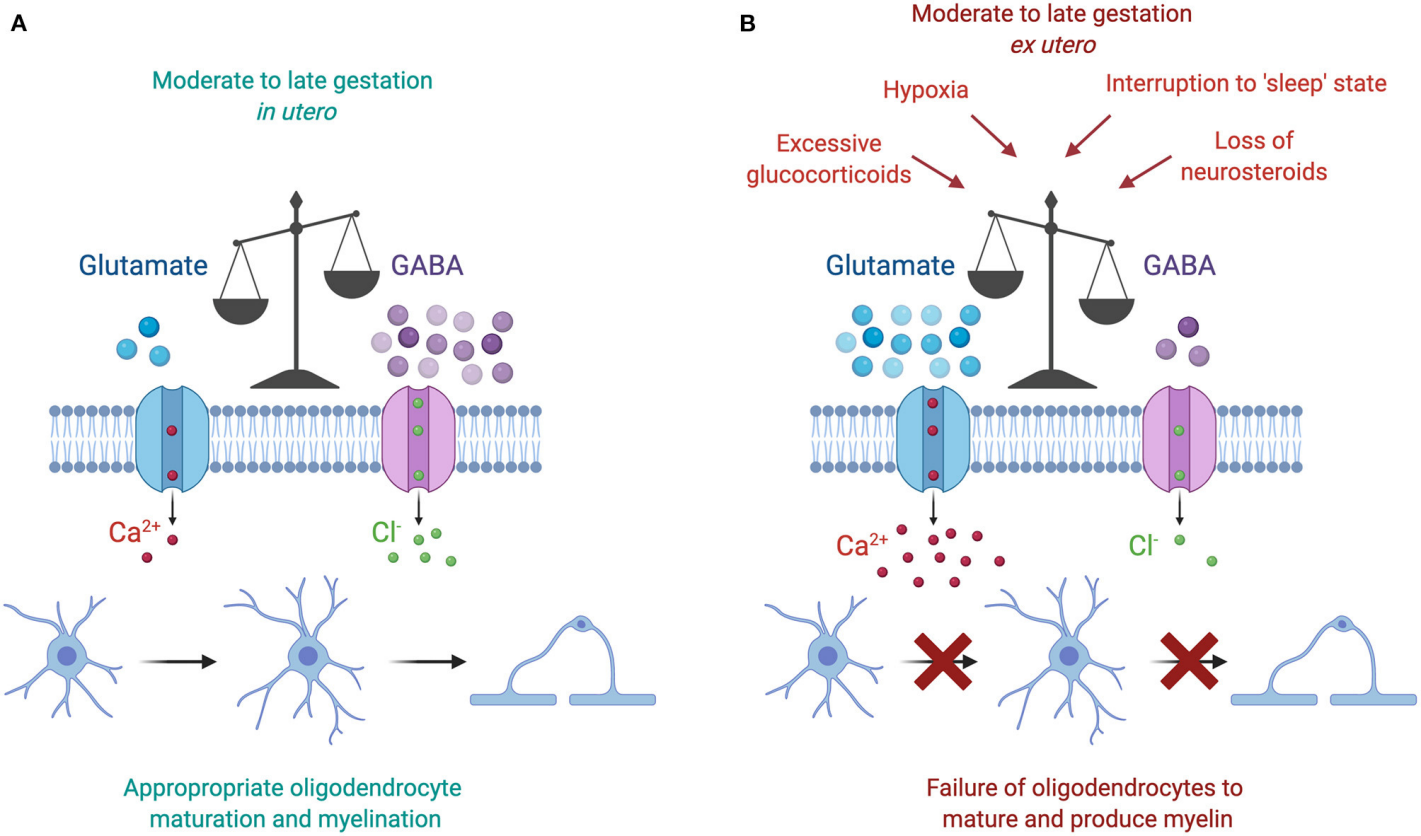
**(A)** The moderate to late gestation *in utero* neural environment is characterized by a dominance of GABAergic activity, which plays a key role in promoting oligodendrocyte maturation and myelination. **(B)** The preterm *ex utero* environment is characterized by a loss of GABAergic activity following a reduction in placental neurosteroid supply. Subsequently, this loss disrupts the fetal “sleep” state, and may also coincide with additional adverse insults, including excessive glucocorticoid concentrations and periods of hypoxia. The resulting increase in glutamate action increases the amount of calcium ions flowing into the oligodendrocyte, thus preventing its normal development and production of myelin. Figure created with BioRender.com.

It is interesting to note that each of the environmental insults discussed above is associated with a downstream increase in glutamate activity (Figure 3). Briefly, increased cortisol concentrations are linked with reductions in GABAergic neurosteroid interactions and a shift toward a glutamate dominated environment, increased wake and arousal periods are associated with raised glutamate concentrations, and hypoxia triggers a release of intracellular glutamate into the extracellular space. Currently available data regarding glutamate levels in the preterm infant are minimal and conflicting. In preterm infants without signs of brain injury, magnetic resonance imaging of the right frontal lobe at term equivalent age has shown that preterm birth at <27 weeks is associated with lower GABA and glutamate concentrations compared to term controls at 42 weeks post-menstrual age (136). Conversely, a recent prospective study involving preterm infants born <32 weeks showed that glutamate concentrations in the frontal lobe rises with increasing postnatal age, and furthermore that GABA concentrations correlated negatively with increasing gestational age at birth (137). The authors of this study speculated that preterm birth may therefore accelerate neurotransmitter production prematurely after early exposure to extra-uterine stresses (137). Thus, based on the most recent study increasing glutamate in the postnatal period may play a key role in the failure to myelinate following preterm birth.

### Increasing GABAergic Action to Prevent Glutamate Excitotoxicity

**Figure 4:**
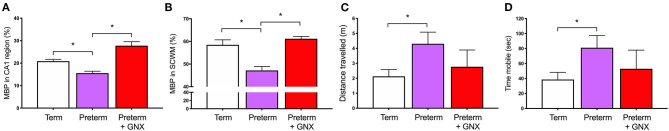
Ganaxolone (GNX) 5 mg/kg by subcutaneous injection daily for 1 week following preterm birth restores mature myelin coverage in **(A)** the CA1 region of the hippocampus, and **(B)** the overlying subcortical white matter at childhood-equivalent age (corrected postnatal day 28). Behavior was also restored toward a term born phenotype for **(C)** the distance traveled, and **(D)** the time spent mobile in the open field arena. (*p < 0.05, *n* = 4–10). Adapted from (55).

The second half of gestation is a period of dramatic changes in the GABAergic system, with the density of GABAergic neurons peaking at full term and the migration of GABAergic neurons occurring throughout mid-gestation and into the early postnatal period. Therefore, preterm birth occurs at a vulnerable developmental window where the GABAergic system is not yet fully matured and ready for exposure to the *ex utero* environment. As discussed above, there is substantive evidence that overactivation of glutamate receptors on oligodendrocytes increases calcium ion flow into the cell, resulting in activation of cell death pathways. In the preterm neonate, we propose that targeting the immature GABAergic system by increasing action on GABA_A_ receptors may prevent this damaging excitatory input and create a normal balance of inhibition:excitation. Organotypic cerebellar slice studies show that targeting the GABA_A_ receptor using allopregnanolone or GABA itself promotes oligodendrocyte maturation and the production of myelin (26, 64, 65, 138, 139). Furthermore, drugs that act by increasing the available concentration of GABA either by preventing it's metabolism (Vigabatrin) or by inhibiting its' reuptake into astrocytes (Tiagabine) are able to increase the development of mature oligodendrocytes following hypoxia-mediated depletion (26). Seemingly, this restoration of the lineage is due to the action of GABA on oligodendrocyte GABA_A_ receptors, as blocking these receptors using Bicuculline prevents the improvement in maturation (26). We have also demonstrated *in vivo* that increasing stimulation of GABA_A_ receptors with GABA_A_ receptor agonists following perinatal compromise is associated with an increase in myelination, and importantly a return to normal behavioral outcomes. In our model of prenatal stress, myelination deficits seen in juvenile guinea pigs exposed to stress *in utero* were restored by increasing neurosteroidogenic capacity in the week following spontaneous term birth (140). Furthermore, and highly pertinent to the search for potential therapies, we were also able to restore normal behavior and oligodendrocyte development in guinea pig offspring born premature. Moderately preterm (GA62; term = GA69) guinea pigs were administered the allopregnanolone analog Ganaxolone in the week following birth, resulting in increased myelination in the hippocampus and overlying subcortical white matter and a normal behavioral phenotype at childhood-equivalent age (Figure 4) (55). These data highlight how restoring inhibitory GABAergic action following insults such as hypoxia, excessive glucocorticoids, and early exposure to the *ex utero* environment can rectify or prevent perturbed oligodendrocyte maturation and ultimately increase the production of myelin. Thus, pharmacologically promoting GABAergic activity in the preterm neonatal brain warrants continued attention to determine feasibility for preventing myelination deficits and improving behavioral outcomes. Furthermore, approaches targeting GABA_A_ receptor specific subtype compositions may allow the selective targeting of appropriate stages of the oligodendrocyte lineage to improve myelination.

## Conclusions

Moderate to late gestation preterm birth is associated with poor neurodevelopmental and behavioral outcomes. Diffuse deficits in myelination that persist into later life are relatively common among those born preterm. Post-mortem and animal studies identify an arrest in oligodendrocyte maturation in the neonatal preterm brain, highlighting a key role of the postnatal environment in oligodendrocyte dysfunction. Environmental insults in the immediate *ex utero* period, such as hypoxia and increased glucocorticoid exposure, have a compounding effect on biological immaturity of the preterm neonatal brain. The vulnerability of these neonates at disadvantage is further increased by the premature loss of neurosteroids that increase GABAergic action in the developing brain to promote oligodendrocyte maturation and protect these cells from damaging insults. There appears to be a common downstream effect of these events that involves increases in extracellular glutamate and an overactivation of glutamate receptors on oligodendrocytes. This in turn results in a failure of oligodendrocytes to mature and produce myelin. Preclinical studies suggest that increasing GABAergic action, and thereby dampening the effect of glutamate, may enable oligodendrocytes to mature despite adverse events. Therefore, increasing GABAergic action in the immediate neonatal period may be a feasible avenue for targeted therapy following preterm birth to prevent myelination deficits and subsequent poor behavioral outcomes.

## Author Contributions

JS and JH were primary authors of the review. GC and HP assisted with writing and provided edits. All authors contributed to the article and approved the submitted version.

## Conflict of Interest

The authors declare that the research was conducted in the absence of any commercial or financial relationships that could be construed as a potential conflict of interest.
